# Peripheral Lipid Signatures, Metabolic Dysfunction, and Pathophysiology in Schizophrenia Spectrum Disorders

**DOI:** 10.3390/metabo14090475

**Published:** 2024-08-28

**Authors:** Sally Wu, Kristoffer J. Panganiban, Jiwon Lee, Dan Li, Emily C.C. Smith, Kateryna Maksyutynska, Bailey Humber, Tariq Ahmed, Sri Mahavir Agarwal, Kristen Ward, Margaret Hahn

**Affiliations:** 1 Schizophrenia Division, Centre for Addiction and Mental Health, Toronto, ON M6J 1H3, Canadatariqb.ahmed@mail.utoronto.ca (T.A.);; 2 Institute of Medical Sciences, University of Toronto, Toronto, ON M5T 1R8, Canada; 3 Department of Psychiatry, University of Toronto, Toronto, ON M5T 1R8, Canada; 4 Banting and Best Diabetes Centre, University of Toronto, Toronto, ON M5G 2C4,Canada; 5 Clinical Pharmacy Department, College of Pharmacy, University of Michigan, Ann Arbor, MI 48109, USA; 6 Department of Pharmacy, Michigan Medicine Health System, Ann Arbor, MI 48109, USA

**Keywords:** schizophrenia, psychosis, lipids, lipidomics, antipsychotics, metabolic dysfunction

## Abstract

Metabolic dysfunction is commonly observed in schizophrenia spectrum disorders (SSDs). The causes of metabolic comorbidity in SSDs are complex and include intrinsic or biological factors linked to the disorder, which are compounded by antipsychotic (AP) medications. The exact mechanisms underlying SSD pathophysiology and AP-induced metabolic dysfunction are unknown, but dysregulated lipid metabolism may play a role. Lipidomics, which detects lipid metabolites in a biological sample, represents an analytical tool to examine lipid metabolism. This systematic review aims to determine peripheral lipid signatures that are dysregulated among individuals with SSDs (1) with minimal exposure to APs and (2) during AP treatment. To accomplish this goal, we searched MEDLINE, Embase, and PsychINFO databases in February 2024 to identify all full-text articles written in English where the authors conducted lipidomics in SSDs. Lipid signatures reported to significantly differ in SSDs compared to controls or in relation to AP treatment and the direction of dysregulation were extracted as outcomes. We identified 46 studies that met our inclusion criteria. Most of the lipid metabolites that significantly differed in minimally AP-treated patients vs. controls comprised glycerophospholipids, which were mostly downregulated. In the AP-treated group vs. controls, the significantly different metabolites were primarily fatty acyls, which were dysregulated in conflicting directions between studies. In the pre-to-post AP-treated patients, the most impacted metabolites were glycerophospholipids and fatty acyls, which were found to be primarily upregulated and conflicting, respectively. These lipid metabolites may contribute to SSD pathophysiology and metabolic dysfunction through various mechanisms, including the modulation of inflammation, cellular membrane permeability, and metabolic signaling pathways.

## 1. Introduction

Schizophrenia spectrum disorders (SSDs) represent a series of chronic mental illnesses affecting approximately 1% of the world’s population [1]. Beyond functional impairment caused by the illness itself, patients with SSDs are at increased risk of suffering from obesity, dyslipidemia, and type 2 diabetes [2]. These metabolic comorbidities contribute to a 2–3-fold increased risk of developing cardiovascular disease and a decreased lifespan of 15–20 years in SSDs compared to the general population [3,4,5].

Metabolic comorbidities in SSDs are understood to be intrinsic to the disease itself and further worsened over time by antipsychotic (AP) medications and lifestyle factors. For example, in AP-naive patients with SSDs, in whom the confounding effects of APs and illness duration are minimal, disturbances in glucose homeostasis and lipid profiles are already observed [5,6,7]. However, over time, lifestyle and social factors including inactivity, dietary habits, and reduced access to medical care contribute to the progressive worsening of metabolic comorbidity throughout the illness [8,9]. Finally, APs are associated with well-established significant metabolic side effects including lipid abnormalities, glucose dysregulation, and weight gain [5,8,10,11,12].

While the exact mechanisms underlying the pathophysiology of SSDs, intrinsic metabolic dysfunction, and AP-induced metabolic dysfunction are unknown, the role of lipid metabolism has been implicated in multiple studies across different stages of illness. In particular, states of dyslipidemia, adiposity, and insulin resistance, which are observed in both AP-naive and AP-treated patients with SSDs, have been associated with altered lipid metabolism [13]. In terms of potential mechanisms, phospholipids are an important component of neuronal membranes. As such, abnormalities in brain phospholipid metabolism can alter neuronal and hence brain morphology, and this has been suggested to underly the pathophysiology of SSDs [14].

Lipidomics is a high-throughput approach that allows for the comprehensive characterization of lipid metabolism. This is achieved through analyzing lipid metabolites in biological samples. Currently, mass spectrometry (MS)-based techniques are the most powerful tools available for detecting, identifying, and quantifying lipid metabolites [15]. Furthermore, MS-based techniques can also be divided into targeted and untargeted approaches, with targeted lipidomics aiming to quantify specific lipid species and untargeted lipidomics examining all detectable lipid metabolites [16]. When applied to studying disease states, lipidomics can lead to the identification of dysregulated lipid metabolites, also known as lipidomic signatures. These lipidomic signatures can facilitate the discovery of biomarkers and underlying pathophysiological mechanisms [17,18].

Over the past decade, a growing number of studies have applied lipidomic tools in SSDs. Lipidomic signatures identified in studies may provide clues to candidate biomarkers and underlying associations of pathophysiology and metabolic dysfunction within SSDs. In particular, the application of lipidomics to minimally AP-treated patients can help to elucidate metabolic and pathophysiological dysregulations that are present without the confounding effects of AP treatment. Therefore, through a systematic search, this review aims to (1) identify peripheral lipidomic signatures observed in minimally AP-treated patients and (2) investigate the impact of APs on lipidomic signatures by comparing AP-treated patients to healthy controls (HCs) or by comparing pre-to-post AP treatment in patients.

## 2. Materials and Methods

This study was conducted in accordance with the preferred reporting items for systematic reviews and meta-analyses (PRISMA) methodology and reporting standard. A protocol for this review was submitted to the PROSPERO international database of prospectively registered systematic reviews (PROSPERO #CRD42022298057).

### 2.1. Search Strategy

Studies were identified through a systematic search. MEDLINE, Embase, and PsycINFO databases were searched for relevant peer-reviewed studies published prior to February 2024. The following search string was used: (lipidom* OR metabolom* OR metabonom*) AND (schizophrenia OR schizophreniform OR schizoaffective OR psychosis OR antipsychotic*). The search string details are provided in Supplementary Table S1. The search was limited to studies written in English and conducted on humans.

### 2.2. Study Selection

Study selection and de-duplication took place in Covidence. The resulting articles were first screened based on title and abstract, followed by full-text screening by six reviewers (S.W., J.L., K.J.P., D.L., E.C.C.S., and K.M.). Agreement from a minimum of two authors was required for a study to be included or excluded. Disagreements were resolved between all authors by reviewing source papers. Studies were selected based on the following criteria: (1) Study Design: Any clinical trials and observational studies including cohort, case–control, and cross-sectional studies were included. We excluded systematic reviews, meta-analyses, case reports, case series, conference proceedings and abstracts, ideas, editorials, opinions, and animal research studies. While systematic reviews and meta-analyses were excluded from the final review, we mined their reference lists to identify additional references that met the eligibility criteria. (2) Study Population: Populations of interest included individuals diagnosed with SSDs according to a recognized diagnostic criterion (e.g., International Classification of Diseases, ICD; Diagnostic and Statistical Manual of Mental Disorders, DSM). We included both AP-treated and minimally AP-treated patients, where minimally AP-treated was defined as having no prior exposure to APs (AP-naive) or not using APs for more than 3 weeks in the past 3 months (AP-free). We excluded a study if most participants presented with comorbidities or concomitant medications deemed to have a clinically significant impact on metabolic homeostasis such as metformin or antidiabetic drugs [19]. We also excluded post-mortem studies. (3) Comparator: For review question 1, we included studies that identified lipidomic signatures differentiating minimally AP-treated patients from HCs. For review question 2, we included studies that investigated the impact of APs on lipidomic signatures by comparing AP-treated patients with SSDs to HCs or by comparing pre-to-post AP treatment in patients. For both questions, HCs were defined as not having a history of psychiatric illness, no previous AP treatment, and no other medical conditions or use of medications deemed to impact lipid signatures. (4) Outcomes: Included studies were required to assess lipid signatures peripherally. Lipid signatures were defined as lipids measured using lipidomic approaches that were reported to be significantly dysregulated. Lipid signatures that differed from the comparator group across at least two studies for each objective were included in this review. In addition, only studies that reported on the direction of change (up- or downregulated) for each metabolite were included.

### 2.3. Data Extraction

All data were independently extracted and reviewed by five authors (J.L., S.W., K.J.P., D.L., and E.C.C.S.). Lipid metabolites that significantly differed in minimally AP-treated patients and AP-treated patients compared to controls were extracted separately. For each significant metabolite, the direction of change was noted. If an equal number of studies reported a metabolite as being upregulated vs. downregulated, then the overall direction was defined as “conflicting”. For studies that examined a combination of minimally AP-treated, AP-treated, and pre-to-post-treated patients, the outcomes were extracted separately for each group if the data were reported in this manner. Otherwise, if the authors identified lipidomic signatures for both minimally AP-treated and AP-treated patients combined, then the study was classified based on the AP condition group that represented 50% or more of the included participants. One minimally AP-treated study examined male and female participants separately, with no lipidomic investigation across both sexes [20]. As such, for this study, the lipidomic signatures were extracted separately for males and females. In addition to lipidomic signatures, other pertinent information, including author(s), publication year, study population, study design, number of cases, number of controls, sex ratio, age, AP treatment status of patients, types of APs used, analytical tool used, Human Metabolome Database (HMDB) IDs [21], and peripheral tissue analyzed, were also collected.

### 2.4. Risk of Bias Assessment

The Johanna Briggs Institute (JBI) Critical Appraisal Checklist for Case Control Studies and the JBI Critical Appraisal Checklist for Cohort Studies were used to assess bias in the context of our outcomes of interest (i.e., peripheral lipid signatures) [22]. Risk of bias assessments were conducted by four independent reviewers (S.W., D.L., K.J.P., E.C.C.S.), with two individuals assigned per study, and conflicts were resolved through group discussion and consensus.

## 3. Results

**Figure 1:**
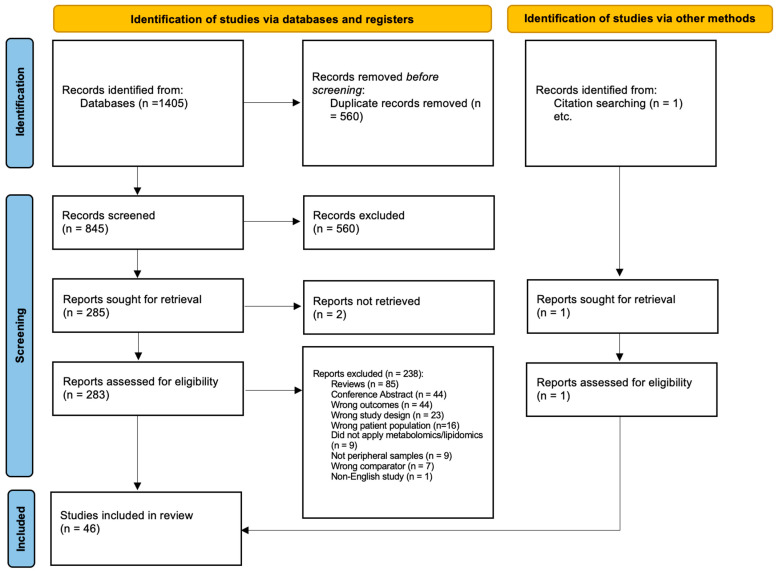
Preferred reporting items for systematic reviews and meta-analyses (PRISMA) flow chart of included studies.

The search identified 1405 records across three databases (Figure 1). Of these, 560 duplicates were removed, resulting in 845 studies that went through title and abstract screening. After title and abstract screening, 560 studies were excluded based on the inclusion and exclusion criteria. Subsequently, 285 studies were assessed for eligibility based on the full text, leading to 240 additional studies being excluded. Thus, a total of 46 studies were included in this review (one study was found during review mining).

Of the 46 included studies, 17 studies investigated lipidomic signatures between minimally AP-treated patients with SSDs and HCs [20,23,24,25,26,27,28,29,30,31,32,33,34,35,36,37,38], 28 studies investigated lipidomic signatures between AP-treated patients and HCs [33,36,39,40,41,42,43,44,45,46,47,48,49,50,51,52,53,54,55,56,57,58,59,60,61,62,63,64], and 13 studies looked at patients pre- vs. post-treatment [20,26,27,29,31,33,34,59,60,62,65,66,67]. Two studies were included in both the minimally AP-treated and AP-treated comparisons as they had sub-groups investigating each aim [33,36]. Additionally, seven studies in the minimally AP-treated group [20,26,27,29,31,33,34] and four studies in the AP-treated group [33,59,60,62] were included in the pre-to-post-treated comparison as there were separate sub-groups for this aim. The most common techniques for lipidomic analysis were liquid chromatography–MS and gas chromatography–MS. A full list of the types of techniques and the participants’ demographic data can be found in Table 1, Table 2 and Table 3. None of the studies included in this review were deemed to be of low quality or have a high risk of bias in domains such as the selection of participants, measurement of outcomes, selection of reported results, and bias due to missing data. Supplemental Tables S2–S4 provide additional characteristics about the included studies such as nicotine use and concomitant medications.

The lipid classes that comprised the altered metabolite signatures in minimally AP-treated patients vs. HCs, AP-treated patients vs. HCs, and pre-to-post AP treatment were primarily glycerophospholipids and fatty acyls. For minimally AP-treated patients, it was observed that the most common class was glycerophospholipids, and they were primarily downregulated, while the most common direction across all the lipid metabolites in this comparison was conflicting (i.e., equal number of upregulated and downregulated). For the lipid signatures in AP-treated patients, the most common metabolite class was fatty acyls, and they had approximately equal numbers in the conflicting and upregulated directions. Interestingly, the downregulated metabolites for this comparison mostly comprised glycerophospholipids. For the pre-to-post AP treatment comparison, there were similar numbers of dysregulated fatty acyls and glycerophospholipids, with fatty acyls predominately being in a conflicting direction and glycerophospholipids being mostly upregulated. Table 4, Table 5 and Table 6 detail the metabolites that appeared at least twice across different studies per comparison and additional study information such as whether a correction for multiple comparisons was applied, significant metabolic indices (i.e., weight, body mass index (BMI), and clinical measures of glucose and cholesterol parameters), and/or symptom scale differences between the comparison groups, if reported.

## 4. Discussion

This review explored alterations in lipid metabolites associated with SSDs and AP use to describe potential associations that may underlie SSD pathophysiology and metabolic dysfunction intrinsic and extrinsic to SSDs. We focused on three comparator groups: minimally AP-treated patients with SSDs vs. HCs, AP-treated patients vs. HCs, and pre-to-post AP treatment.

Glycerophospholipids and fatty acyls were the metabolite classes that were most frequently impacted across the three comparison groups, suggesting a potential role in the pathophysiology of and metabolic dysfunction within SSDs. Glycerophospholipids represent a class of metabolites that are primarily found in the cellular membrane and are involved in membrane stability and fluidity [68]. Ensuring the stability and fluidity of the cellular membrane is integral for normal cell functioning as the cellular membrane is involved in cellular signaling and provides protection for intracellular components [69]. Glycerophospholipids may play a role in glucose metabolism as low membrane fluidity has been linked to impaired insulin signaling [70]. Additionally, glycerophospholipids are linked to brain structure as oral supplementation can increase hippocampal cell morphology [71]. Glycerophospholipids can also contribute to dendritic development, and abnormalities within dendrites have been associated with neuropsychiatric disorders [72,73].

**Figure 2:**
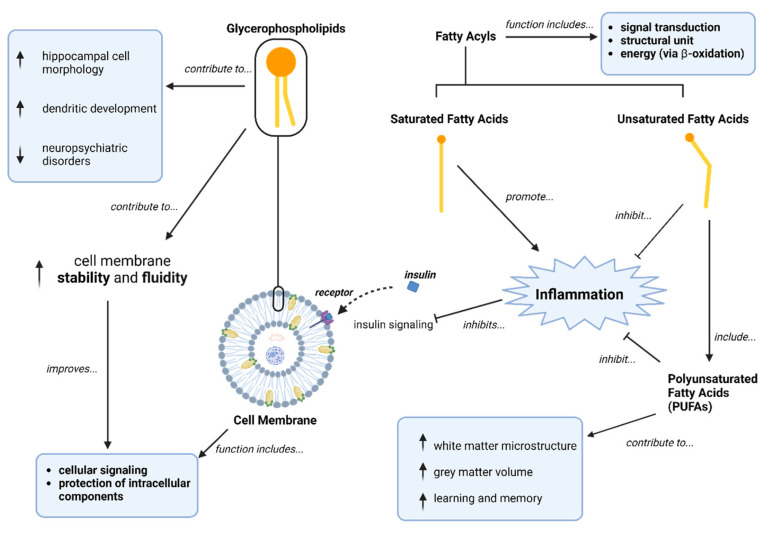
Potential associations between glycerophospholipids, fatty acyls, and schizophrenia spectrum disorders; ↑ = increased, ↓ = decreased.

Fatty acyls have a multitude of functions such as providing energy to cells, mediating signal transduction pathways, and acting as a structural unit in cell membranes [74]. Fatty acids, the largest sub-type of fatty acyls, are important components for metabolism as they provide energy to tissues through β-oxidation. Previous research has shown that morphological brain changes, such as improving white matter microstructure, increasing grey matter volume, and improvements in learning and memory, can occur with polyunsaturated fatty acid (PUFA) supplementation [75,76]. Additionally, fatty acyls can potentially be associated with insulin resistance through inflammation. For example, saturated fatty acids are viewed as pro-inflammatory molecules and inflammation is linked with insulin resistance [77]. Unsaturated fatty acids may play an opposing role in insulin action as they are viewed as anti-inflammatory molecules and may potentially have protective effects [77]. Figure 2 summarizes the potential associations between glycerophospholipids, fatty acyls, and SSDs.

### 4.1. Aim 1: Lipidomic Signatures in Minimally AP-Treated Patients Compared to Healthy Controls

In minimally AP-treated patients with SSDs, it was observed that glycerophospholipids were the most common class impacted, and they were primarily downregulated. This downregulation is consistent with potential mechanisms involved in SSD pathophysiology such as increased phospholipase A2 (PLA2) activity and dendritic spine alterations [78,79]. PLA2s are a family of enzymes that hydrolyze glycerophospholipids, and increased activity of PLA2s is associated with an increased breakdown of the cell membrane [78]. Therefore, lower levels of glycerophospholipids found in minimally AP-treated patients may help explain illness pathophysiology as the breakdown of the cell membrane can impact cell stability and signaling. Dendritic spine alterations have been found in SSDs including reductions in density and arborization [79]. Given that glycerophospholipids are involved in dendritic growth, these alterations may be linked to decreased levels of glycerophospholipids [80]. Additionally, the reduction in dendritic spine density and arborization may help explain structural brain changes reported and/or cognitive deficits observed in SSDs [81,82,83]. As all the studies included in this comparison involved patients that were minimally exposed to APs, the observed downregulation of glycerophospholipids may represent a biomarker intrinsic to SSDs.

Past research has shown that glycerophospholipids may also be associated with metabolic dysfunction as reduced levels have been observed in individuals with impaired fasting glucose and type 2 diabetes [84]. However, this association was not observed in our review as the majority of included studies did not find significant differences in metabolic parameters between patients and HCs. A potential reason for this observation is that most studies only used BMI as a metabolic parameter and the authors may have matched patients and HCs based on this value to reduce potential group differences. As such, critical parameters like fasting glucose and insulin were not collected in most of the studies, making it difficult to identify any differences based on these parameters. The results also showed that the most common direction of change for the lipid metabolites was conflicting, which could potentially reflect the inherent heterogeneity of SSDs.

### 4.2. Aim 2: Investigate the Impact of APs on Lipidomic Signatures by Comparing AP-Treated Patients with SSDs to HCs or by Comparing Pre-to-Post AP Treatment in Patients

In AP-treated patients vs. HCs, a trend within the results was that half of the downregulated metabolites were from the glycerophospholipid class. Interestingly, glycerophospholipids are a major component of mitochondria, and a decrease in levels can lead to an increase in mitochondrial permeability, which has been linked to metabolic disorders [85]. Taken together, the possibility may exist that the observed downregulation in glycerophospholipids could be related to the metabolic dysfunction associated with APs [86,87]. It was also observed that fatty acyls were primarily in the conflicting direction in AP-treated patients. One fatty acyl, nervonic acid, was increased across two studies, and previous research suggests that this change may be related to symptom control in relation to AP treatment. For example, one study found that decreased levels of nervonic acid were associated with the development of psychosis in ultra-high-risk individuals [88] while another found that increases in polyunsaturated fatty acids were associated with symptom improvement. As nervonic acid is a monounsaturated (rather than polyunsaturated) fatty acid, it is possible that the observed association may not hold; however, given the convergence of our results with these findings, future research is warranted. Furthermore, it is difficult to parse out the cumulative effects of lifestyle habits (diet and physical activity), intrinsic and extrinsic metabolic risk, and the effects of the illness itself, which can all have potential effects on lipid metabolites. Differing dietary habits between patients and HCs have been noted. For example, patients have been shown to consume higher total dietary fat and increased caloric intake [89,90]. Research has also shown that the lipidome can be impacted by dietary fat, which may further explain some of the conflicting metabolites. The AP exposure of the patients also varied widely in dose, duration, and type, which may also help to explain the variation in the results [11]. Furthermore, in the studies that found downregulated glycerophospholipids, only a couple of studies [50,61] observed significantly different metabolic parameters; however, similar to the minimally AP-treated aim, most studies only measured BMI.

For the pre-to-post AP-treated patients, fatty acyls and glycerophospholipids were the classes most impacted, with fatty acyls primarily being in a conflicting direction and glycerophospholipids being upregulated. Potential reasons for fatty acyls having a conflicting direction may be due to differing follow-up lengths (2–78 weeks), the specific AP agent, and possibly the AP dose. Additionally, not all studies included AP-naive patients at baseline, and authors often failed to report on the washout periods from any pre-existing AP treatments. Three studies found significant differences in linoleic acid [34,60,62], a PUFA, two of which found that it was upregulated after AP treatment and that it correlated with significant associated improvements in overall and positive symptom scores [60,62]. The latter finding is consistent with previous research demonstrating an association between PUFA deficiency and symptom severity [76,91]. Lysophosphatidylcholines (LPCs), a sub-type of glycerophospholipids, were found to be upregulated across studies post-AP treatment. This is also supported by previous research that identified negative associations between LPCs and symptom severity in SSDs [66].

A few of the studies included in the pre-to-post comparison examined lipid signatures in relation to metabolic outcomes. For example, across two studies examining AP-naive patients at baseline, it was reported that cholesterol ester (22:6) and phosphocholine (38:6) were reduced after AP treatment [31,65], and this occurred in association with weight gain [65]. Additionally, LPCs (14:0), (18:0), (18:1), and (20:3) were found to be upregulated post-AP treatment [27,29,34,59,65,66]. In keeping with these observations, past research has demonstrated that increased levels of these metabolites are associated with weight gain, with LPCs (14:0) representing an independent contributor after a regression analysis [92]. As such, these longitudinal studies provide additional support for the idea that lipid signatures could represent biomarkers linked to AP-induced weight gain [93]. However, once again, differences in AP type, duration, and dose as well as the heterogeneity of SSDs may have had potentially confounding effects on the observed results.

### 4.3. Limitations and Recommendations for Future Studies

There was a paucity of longitudinal studies examining lipid signatures pre-to-post AP treatment, which may have contributed to the conflicting lipidomic signatures observed across studies. Similarly, for many of the differing lipid signatures identified, there were disagreements among studies across the three population groups regarding the direction of dysregulation. The inconsistencies in the direction of dysregulation may also be indicative of the complex mechanisms by which lipid metabolism may influence psychopathology and metabolic dysfunction. As discussed above, factors such as sex, diet, duration of illness, smoking or other substance use, activity levels, and AP type, duration, and dose may impact lipid signatures, and many studies did not account for these [9,94]. For example, very few studies matched their cases and controls based on these factors, which may have confounded the results. Another limitation is that cultural differences may impact the results. Approximately half of the studies were conducted in Western societies while the other half were conducted in Eastern societies. The respective individualistic vs. collectivistic culture may impact the results. For example, greater social support in some societies can be a protective factor and diet can also vary between cultures [95]. In addition, previous research has shown that APs can vary in their metabolic risk, with potentially differential effects on the lipidome [11]. Since the included studies used a variety of different APs, it was challenging to discern how lipid metabolites may be impacted by APs with high vs. low metabolic risk profiles. As such, future studies should control for the differing risk profiles of these medications. Furthermore, the studies included in our review employed a range of lipidomic tools to assess peripheral lipid signatures in our population of interest. As each technique has different advantages and disadvantages, this may influence the selectivity, sensitivity, and accuracy of the observed results. Moreover, most of the included studies used targeted metabolomic techniques, which may limit the number of identifiable lipids. Future studies should also limit the heterogeneity of reportable outcomes by standardizing methods such as fasting blood sample collection and corrections for multiple comparisons.

## 5. Conclusions

In the present review, we demonstrate that certain lipidomic signatures may represent biomarkers related to SSDs, as assessed using minimally AP-treated patients. Specifically, we found that the majority of lipid metabolites were from glycerophospholipids, and they were mostly downregulated. Additionally, we attempted to elucidate the effects of APs on lipid metabolites by comparing AP-treated patients to HCs and pre-to-post AP-treated patients. For AP-treated patients, it was found that the most affected class was fatty acyls, and they were mainly in the conflicting direction. For the pre-to-post AP treatment comparison, there were similar numbers of dysregulated fatty acyls and glycerophospholipids, with fatty acyls mostly being in a conflicting direction and glycerophospholipids being predominantly upregulated. These signatures in turn may be associated with SSD pathophysiology as well as intrinsic and AP-induced metabolic dysfunction through various mechanisms, including the modulation of inflammation, cellular membrane permeability, and metabolic signaling pathways. As such, identifying these lipidomic signatures may aid in the development of better diagnostic tools, possibly leading to novel treatment regimens; however, there is a need for further, well-designed prospective longitudinal studies that assess lipidomic changes in relation to metabolic alterations, AP use, psychopathology, and treatment outcomes.

## Figures and Tables

**Table 1:** Characteristics of cross-sectional components of studies (N = 17) examining minimally antipsychotic-treated schizophrenia spectrum disorder patients.

Author, Year	Analytical Tool	Diagnosis	AP-Naive Window	AP-Naive			Controls			Significantly Different Metabolic Parameters (SSDs Compared to HCs)
				N	Sex, Female (%)	Mean Age (SD)	N	Sex, Female (%)	Mean Age (SD)	
Bicikova 2013 # [20]	GC–MS	SSDs	AP-naive	21	38	Males: Median = 31 (range: 22–52) Females: Median = 35 (range: 24–52)	32	41	Males: Median = 35 (range: 23–53) Females: Median = 35 (range: 23–51)	TG *: ↑ Cholesterol *: ↑ HDL *: ↓ * Only in AP-naive FEP males compared to HCs
Cai 2012 [23]	UPLC–MS/MS and H-NMR	SSDs	AP-naive	11	45.45	27.6 (9.5)	11	45.45	27.6 (9.5)	FBG: ↓ Insulin: ↑
Cui 2021 [24]	UPLC–QTOF-MS/MS	FES	AP-naive	83	42.2	25.60 (7.42)	78	50	23.03 (6.45)	NR
Kaddurah-Daouk 2012 [25]	GC + flame ionization detector	SSDs	AP-naive	20	35	27.0 (9.8)	17	66	37.9 (9.1)	None
Kriisa 2017 # [26]	FIA–MS/MS and LC–MS	FEP, SSDs	AP-naive	38	NR	Range: 18–45	37	NR	NR	None
Lee 2023 [32]	LC–MS	PSDs	AP-naive or AP-free (3 weeks or less in past 3 months)	25	32	21.8 (4.1)	6	50	25.2 (3.5)	None
Leppik 2020 # [27]	FIA–MS/MS and LC–MS	FEP; SSDs	AP-naive	53	39.60	26.2 (6.0)	37	56.80	24.8 (5.3)	NR
Liu 2014 [38]	GC–MS	FES and SSDs	AP-naive (42.2% AP-treated)	45	60	33.22 (12.9)	50	56.00	37.26 (8.67)	NR
Liu 2021 [28]	LC–MS-based multiple reaction monitoring	SSDs	AP-naive	38	57.9	34.82 (13.33)	25	40	33.7 (12.5)	None
Qiao 2016 # [29]	LC–MS	FES	AP-naive	15	100.00	28.25 (5.34)	15	100	27.6 (6.3)	None
Schwarz 2011 [30]	LC–MS	FEP, SSDs	AP-naive	70	34.20	29.3 (10.0)	59	44.1	27.5 (5.9)	NR
Shang 2022 *^,^# [33]	UPLC–TOF-MS	FEP	Not treated for more than 30 days; mean (SD): 10 (2.9) days	25	48	31.40 (1.96) ^a^	21	62	25.81 (1.34) ^a^	None
Song 2023 # [34]	UPLC–QTOF-MS	SSDs	AP-naive	43	42	27.3 (6.0)	29	55	27.1 (4.2)	None
Su 2023 [37]	FIA/LS–MS	SSDs	AP-naive	60	72	38.08 (10.48)	36	56	38.03 (9.66)	BMI: ↓
Wang X 2024 * [36]	FIA–MS/MS and LC–MS/MS	SSDs	AP-naive	38	100	39.74 (9.83)	19	100	40 (9.1)	None
Wang Z 2024 [35]	LC–MS	SSDs	AP-naive	127	52.8	21.65 (7.55)	92	62	22.95 (2.59)	None
Yan 2018 # [31]	LC–MS	SSDs	AP-naive	20	45.00	32.1 (9.1)	29	37.9	32.4 (7.8)	NR

a = standard error of the mean; AP = antipsychotic; BMI = body mass index; FBG = fasting blood glucose; FIA = flow injection analysis; FEP = first-episode psychosis; FES = first-episode schizophrenia; GC = gas chromatography; HDL = high-density lipoprotein; H-NMR = proton nuclear magnetic resonance; LC = liquid chromatography; MS = mass spectrometry; MS/MS = tandem mass spectrometry; NR = not reported; PSDs = psychotic spectrum disorders; QTOF = quadrupole time-of-flight; SD = standard deviation; SSDs = schizophrenia spectrum disorders; TG = triglycerides; UPLC = ultra performance liquid chromatography; ↑ = increased; ↓ = decreased; * = shared studies between minimally AP-treated and AP-treated groups; # = shared studies between minimally AP-treated and pre-to-post AP treatment groups.

**Table 2:** Characteristics of cross-sectional components of studies (N = 28) examining antipsychotic-treated patients.

Author, Year	Analytical Tool	Antipsychotics	Diagnosis	Antipsychotic-Treated Patients			Controls			Significantly Different Metabolic Parameters (SSDs Compared to HCs)
				N	Sex, Female (%)	Mean Age (SD)	N	Sex, Female (%)	Mean Age (SD)	
AlAwam 2015 [39]	GC–MS	ARI, CLO, CLZ, FLU, HAL, LEV, MEL, OLA, PAL, PRO, QUE, RIS, ZUC	SSDs	26	23.1	37.27 (12.4)	26	23.1	37.0 (10.7)	NR
Avigdor 2021 [40]	UHPLC–MS	ARI, CLZ, LUR, QUE, RIS, TRI, ZIP	SSDs	31	38.7	23.77 (4.4)	35	45.7	23.74 (3.0)	NR
Campeau 2022 [41]	LC–MS	RIS	SSDs	54	53.7	53.8 (10.0)	51	49	52.1 (11.5)	NR
Cui 2020 [42]	LC–MS	NR	SSDs	54	66.6	38.4 (11.6)	54	75.9	33.6 (9.87)	None
Dickens 2020 [43]	LC–QqQMS	ARI, OLA, PER, RIS	SSDs	8	0	26.4 (3.6)	10	0	27.18 (5.9)	None
Du 2021 [44]	UPLC–MS	NR	SSDs	78	14.10	31.7 (7.7)	66	42.40	24.5 (5.1)	NR
Fukushima 2014 [45]	HPLC–MS	ARI, BLO, FLU, LEV, OLA, PAL, QUE, RIS, SUL,	SSDs	25	56	28.2 (4.4)	27	55.6	26.5 (5.6)	BMI: ↑
He 2012 [46]	LC–MS	AMI, CLZ, HAL, OLA, QUE, RIS	SSDs	213	38.00	36.9 (11.7)	216	48.10%	38.9 (10.6)	None
Kaddurah-Daouk 2007 # [59]	GC + flame ionization detector	ARI, OLA, RIS	SSDs	27	85.1	32.3 (5.0)	16	Matched	Matched	None
Koike 2014 [47]	CE–TOF-MS	NR	SSDs	18	27.78	23.2 (5.4)	14	21.43	25.7 (6.1)	NR
Li 2022 # [60]	GC–MS	ARI, AMI, CLZ, OLA, RIS, Others	SSDs	327	55	32.70 (10.40)	159	52.20	32.86 (10.09)	None
Liu 2020 [48]	LC–TOF-MS and H-NMR	NR	SSDs	55	63.6	33.4 (12.8)	57	63.2	44.4 (13.5)	TC: ↓ TG: ↓ HDL: ↓
Mednova 2021 [49]	LC–MS/MS	NR	SSDs	37	48.65	Median: 35 (IQR: 31.00. 39.00)	36	38.89	Median: 32.5 (IQR: 28.75; 40.25)	None
Oresic 2011 [50]	UPLC–MS	Atypical and typical APs	SSDs	45	57.8	53.7 (12.9)	45	57.8	53.7 (12.9)	T2D (n): ↑ FBG: ↑ TG: ↑ Insulin: ↑ HOMA-IR: ↑ WC: ↑ HDL: ↓
Oresic 2012 [51]	LC–TOF-MS	Atypical APs	SSDs	19	68.4	Median: 51 (IQR: 46.4, 55.6)	34	70.6	Median: 53.4 (IQR: 50.2, 56.6)	BMI: ↑
Paredes 2014 [52]	LC–MS	ARI, CLZ, OLA. QUE, RIS, ZIP	SSDs	60	23.3	42.5 (2.6)	20	30	41.1. (2.6)	Insulin: ↑
Parksepp 2022 [64]	(FIA)−MS/MS + (LC)−MS/MS	Various APs, types NR	FEP	38	57	31.8 (5.9)	58	56	24.7 (4.5)	NR
Qing 2022 [63]	UPLC–MS/MS	NR	SSDs	59	61	37.48 (11.80)	60	58	36.80 (9.74)	None
Shang 2022 *^,^# [33]	UPLC–TOF-MS	ARI, CLZ, HAL, OLA, QUE, RIS	FEP	25	48	31.40 (1.96) ^a^	21	62	25.81 (1.34) ^a^	None
Tasic 2017 [53]	H-NMR	OLA, QUE	SSDs	27	37	36 (10.3)	26	65.4	36 (13.1)	NR
Tasic 2019 [54]	H-NMR	CLZ, HAL, OLA, QUE, RIS	SSDs	50	48	35.4 (9.5)	60	70	36 (10.5)	NR
Tessier 2016 [55]	LC–MS	AMI, ARI, CLZ, CPZ, FLU, HAL, OLA, RIS, SER	SSDs	74	35.2	43.8 (9.3)	40	40	42.6 (13.2)	NR
Wang 2021 [61]	LC–MS	NR	SSDs	119	56.30	29.0 (IQR: 25, 33.3)	109	66.1	30 (IQR: 26, 33)	TC: ↑
Wang 2022 [56]	LC–MS and H-NMR	NR	SSDs	64	NR	44.56 (9.53)	40	NR	43.76 (13.87)	NR
Wang X 2024 * [36]	FIA–MS/MS and LC–MS/MS	OLA, PAL, RIS	SSDs	38	100	39.74 (9.83)	19	100	40 (9.1)	None
Wood 2015 [57]	MS/MS	Atypical APs	SSDs	23	21.7	Median: 47 (range: 25–66)	27	66.7	Median: 47 (range: 25–65)	None
Xuan 2011 # [62]	GC–MS	NR	SSDs	18	44.4	41.3 (16.1)	18	44.4	41 (15.0)	NR
Yang 2017 [58]	UPLC–QTOF-MS	NR	SSDs	60	60	37.2 (12.0)	61	59	36.9 (9.7)	BMI: ↓

a = standard error of the mean; AMI = amisulpride; ARI = aripiprazole; BLO = blonanserin; BMI = body mass index; CE = capillary electrophoresis; CLO = chlorprothixene; CLZ = clozapine; CPZ = chlorpromazine; FBG = fasting blood glucose; FEP = first-episode psychosis; FLU = flupentixol; GC = gas chromatography; HAL = haloperidol; HDL = high-density lipoprotein; H-NMR = proton nuclear magnetic resonance; HPLC = high performance LC; HOMA-IR = Homeostatic Model Assessment for Insulin Resistance; IQR = interquartile range; LC = liquid chromatography; LEV = levomepromazine; LUR = lurasidone; MEL = melperone; MS = mass spectrometry; MS/MS = tandem mass spectrometry; NR = not reported; OLA = olanzapine; PAL= paliperidone; PER= perphenazine; PRO = promethazine; QqQMS = quadrupole tandem MS; QTOF = quadrupole time-of-flight; QUE = quetiapine; RIS = risperidone; SD = standard deviation; SER = sertindole; SSDs = schizophrenia spectrum disorders; SUL = sulpiride; TC = total cholesterol; TG = triglycerides; TOF-MS = time-of-flight mass spectrometry; TRI = trifluoperazine; T2D = type 2 diabetes; UHPLC = ultra-high performance liquid chromatography; UPLC = ultra-performance liquid chromatography; WC = waist circumference; ZIP = ziprasidone; ZUC = zuclopenthixol; ↑ = increased; ↓ = decreased; * = shared studies between minimally AP-treated and AP-treated groups; # = shared studies between AP-treated and pre-to-post AP treatment groups.

**Table 3:** Characteristics of included studies (N = 13) examining pre-to-post antipsychotic-treated patients.

Author, Year; Duration Treated	Analytical Tool	AP-Naive at Baseline	Diagnosis; AP Type	SSD Group			Significantly Different Metabolic Parameters (Post-Treatment Compared to Pre-Treatment)	Significantly Different Symptom Scale Scores (Post-Treatment Compared to Pre-Treatment)
				N	Sex, Female (%)	Mean Age (SD)		
Bicikova 2013 *; 26 weeks [20]	GC–MS	AP-naive	SSDs; AMI, OLA, RIS	22	9	Males: Median 31 (range: 22–52) Females: Median 35 (range: 24–52)	NR	NR
Cao 2019; 8 weeks [67]	LC–MS	AP-naive or no AP use for 30 days	SSDs; ARI, CLZ, CPZ, HAL, OLA, PRO, QUE, RIS, SUL, ZIP	122	57.4	28.91 (6.21)	BMI: ↑ WC: ↑ TG: ↑ VLDL: ↑ FBG: ↓ HDL: ↓	PANSS Total: ↓ PANSS Positive: ↓ PANSS Negative: ↓ PANS General: ↓
Kaddurah-Daouk 2007 #; 2–3 weeks [59]	GC + flame ionization detector	No AP treatment for at least 3 weeks	SSDs; ARI, OLA, RIS	27 (ARI = 4, OLA = 14, RIS = 9)	85.1	32.3 (5.0)	NR	NR
Kriisa 2017 *; 30 weeks [26]	FIA–MS/MS and LC–MS	AP-naive	SSDs; ARI, CLZ, OLA, QUE, RIS, SER, ZIP	36	NR	Range: 18–45	BMI: ↑	PANSS Total: ↓ PANSS Positive: ↓ PANSS Negative: ↓ PANSS General: ↓
Leppik 2020 *; 30 weeks [27]	FIA–MS/MS and LC–MS	AP-naive	FEP, SSDs; ARI, CLZ, OLA, PER, QUE, RIS, SER, ZIP	44	39.60	26.20 (6.00)	BMI: ↑	BPRS: ↓
Li 2022 #; 4 weeks [60]	GC–MS	No AP use for 30 days	SSDs; ARI, AMI, CLZ, OLA, RIS	327	55	32.70 (10.40)	NR	PANSS Total: ↓ PANSS Positive: ↓ PANSS General: ↓
Liu 2021; 4 weeks [66]	UPLC–QTOF-MS/MS	AP-naive	FEP, SSDs; OLA	25	100	27.4 (7.6)	NR	PANSS Total: ↓ PANSS Positive: ↓ PANSS General: ↓
Qiao 2016 *; 4 weeks [29]	LC–MS	AP-naive	FES; OLA	15	100	28.20 (5.34)	LDL: ↑	PANSS Total: ↓ PANSS Positive: ↓ PANSS General: ↓
Qiu 2023; 8 weeks [65]	LC–MS/MS and (FIA–MS/MS	AP-naive	SSDs; RIS	30	50	36.40 (12.10)	Weight: ↑ BMI: ↑ WC: ↑ HC: ↑	PANSS Total: ↓ PANSS Positive: ↓ PANSS Negative: ↓ PANSS General: ↓
Shang 2022 *^,^#; 78 weeks [33]	UPLC–TOF-MS	AP-naive	FEP; ARI, CLZ, HAL, OLA, QUE	25	48	31.40 (1.96) ^a^	NR	PANSS Total: ↓ PANSS Positive: ↓ PANSS General: ↓
Song 2023 *; 4–6 weeks [34]	UPLC–MS	AP-naive	SSDs; OLA	43	42	27.30 (6.00)	BMI: ↑ TG: ↑	PANSS Total: ↓ PANSS Positive: ↓ PANSS Negative: ↓ PANSS General: ↓
Xuan 2011 #; 8 weeks [62]	GC–MS	Unmedicated (duration not specified)	SSDs; RIS	18	44.4	41.3 (16.1)	NR	PANSS Total: ↓ PANSS Positive: ↓ PANSS Negative: ↓
Yan 2018 *; 8 weeks [31]	LC–MS	AP-naive	SSDs; CLZ, HAL, OLA, QUE, RIS	20	45.00	32.1 (9.1)	NR	NR

a = standard error of the mean; AMI = amisulpride; ARI = aripiprazole; BMI = body mass index; BPRS = Brief Psychiatric Rating Scale; CLZ = clozapine; CPZ = chlorpromazine; FBG = fasting blood glucose; FEP = first-episode psychosis; FES = first-episode schizophrenia; FIA = flow injection analysis; GC = gas chromatography; HAL = haloperidol; HDL = high-density lipoprotein; LC = liquid chromatography; LDL = low-density lipoprotein; MS = mass spectrometry; MS/MS = tandem mass spectrometry; NR = not reported; OLA = olanzapine; PAL= paliperidone; PANSS = Positive and Negative Syndrome Scale; PER = perphenazine; PRO = promethazine; QTOF = quadrupole time-of-flight; QUE = quetiapine; RIS = risperidone; SD = standard deviation; SER= sertindole; SSDs = schizophrenia spectrum disorders; SUL = sulpiride; TG = triglycerides; TOF-MS = time-of-flight mass spectrometry; UPLC = ultra-performance liquid chromatography; VLDL = very low-density lipoprotein; WC = waist circumference; ZIP = ziprasidone; ↑ = increased; ↓ = decreased; * = shared studies between minimally AP-treated and pre-to-post AP treatment groups; # = shared studies between AP-treated and pre-to-post AP treatment groups.

**Table 4:** Minimally antipsychotic-treated patients’ lipid signatures that appeared in at least two studies when compared to healthy controls.

Class	Lipid, HMDB ID	Bicikova 2013 [20]	Cai 2012 [23]	Cui 2021 [24]	Kaddurah-Daouk 2012 [25]	Kriisa 2017 [26]	Lee 2023 [32]	Leppik 2020 [27]	Liu 2021 [28]	Qiao 2016 [29]	Song 2023 [34]	Su 2023 [37]	Wang X 2024 [36]	Wang Z 2024 [35]	Yan 2018 [31]	Overall Direction
Fatty Acyls	Stearic acid, HMDB0000827										**-**			**-**		Downregulated
Glycerophospholipids	LPC (14:0), HMDB0010379									**-**					**-**	Downregulated
Glycerophospholipids	LPC (18:0), HMDB0010384										**-**	**-**			**-**	Downregulated
Glycerophospholipids	PC (O-34:2), HMDB0011151							**-**	**-**							Downregulated
Glycerophospholipids	PC (36:2), HMDB0000593							**-**				**-**				Downregulated
Glycerophospholipids	PC (O-36:0), HMDB0013406							**-**				**-**				Downregulated
Glycerophospholipids	PC (O-36:3), HMDB0013425							**-**				**-**				Downregulated
Fatty Acyls	Arachidonic acid, HMDB0001043						**+**		**-**							Conflicting
Fatty Acyls	Behenic acid, HMDB0000944						**+**				**-**					Conflicting
Fatty Acyls	Fumarylcarnitine, HMDB0013134			**-**		**+**										Conflicting
Glycerophospholipids	PC (32:1), HMDB0007872							**-**	**+**							Conflicting
Steroids and Steroid Derivatives	Glycohyocholic acid, HMDB0240607										**-**		**+**			Conflicting
Steroids and Steroid Derivatives	DHEAS, HMDB0001032	**+**									**-**					Conflicting
Glycerophospholipids	LPC (16:0), HMDB0010382		**+**						**+**		**-**					Upregulated
Steroids and Steroid Derivatives	CE 16:1, HMDB0000658											**+**			**+**	Upregulated
Steroids and Steroid Derivatives	CE 20:3, HMDB0006736												**+**		**+**	Upregulated
Steroids and Steroid Derivatives	Cholic acid, HMDB0000619											**+**	**+**			Upregulated

CE = cholesterol ester; DHEAS = dehydroepiandrosterone sulfate; LPC = lysophosphatidylcholine; PC = phosphatidylcholine.

**Table 5:** Antipsychotic-treated patients’ lipid signatures that appeared in at least two studies when compared to healthy controls.

Class	Lipid, HMDB ID	AlAwam 2015 [39]	Avigdor 2021 [40]	Campeau 2022 [41]	Cui 2020 [42]	Dickens 2020 [43]	Du 2020 [44]	Fukushima 2014 [45]	He 2012 [46]	Kaddurah-Daouk 2007 [59]	Li 2022 [60]	Liu 2020 [48]	Mednova 2021 [49]	Oresic 2011 [50]	Oresic 2012 [51]	Paredes 2014 [52]	Parksepp 2022 [64]	Qing 2022 [63]	Tessier 2016 [55]	Wang 2021 [61]	Wang 2022 [56]	Wang X 2024 [36]	Wood 2015 [57]	Xuan 2011 [62]	Yang 2017 [58]	Overall Direction
Fatty Acyls	Arachidonic acid, HMDB0001043				**+**	**-**		**-**			**-**									**-**					**+**	Downregulated
Fatty Acyls	Docosahexaenoic acid, HMDB0002183										**-**												**-**			Downregulated
Fatty Acyls	Linoleic acid, HMDB0000673							**-**			**-**	**-**												**-**		Downregulated
Fatty Acyls	Oleic acid, HMDB0000207	**-**			**+**						**-**			**-**						**+**				**-**	**+**	Downregulated
Fatty Acyls	Palmitic acid, HMDB0000220				**-**						**-**									**+**				**-**	**+**	Downregulated
Fatty Acyls	Stearic acid, HMDB0000827		**+**								**-**			**-**						**+**				**-**		Downregulated
Glycerophospholipids	LPC (16:0), HMDB0010382				**-**										**-**					**-**	**-**					Downregulated
Glycerophospholipids	LPC (17:0), HMDB0012108																			**-**	**-**					Downregulated
Glycerophospholipids	LPC (18:0), HMDB0010384									**-**					**-**					**-**	**-**					Downregulated
Glycerophospholipids	LPE (18:0), HMDB0011130																		**-**	**-**						Downregulated
Glycerophospholipids	LPE (18:1), HMDB0011506																		**-**	**-**						Downregulated
Glycerophospholipids	PC (O-38:6), HMDB0013409								**-**											**-**						Downregulated
Fatty Acyls	9-Hydroxylinoleic acid, HMDB0062652				**+**											**-**										Conflicting
Fatty Acyls	Acetylcarnitine, HMDB0000201				**-**												**+**									Conflicting
Fatty Acyls	Acylcarnitine C16-OH, N/A												**-**				**+**									Conflicting
Fatty Acyls	Acylcarnitine C16:1, N/A												**-**				**+**									Conflicting
Fatty Acyls	Acylcarnitine C16:1-OH, N/A												**-**				**+**									Conflicting
Fatty Acyls	Acylcarnitine C18:1-OH, N/A												**-**				**+**									Conflicting
Fatty Acyls	Arachidic acid, HMDB0002212	**-**																		**+**						Conflicting
Fatty Acyls	Heptadecenoic acid, HMDB0002259	**-**																		**+**						Conflicting
Fatty Acyls	Dihomo-gamma-linolenic acid, HMDB0002925										**-**														**+**	Conflicting
Fatty Acyls	Docosapentaenoic acid, HMDB0006528										**-**														**+**	Conflicting
Fatty Acyls	Oleamide, HMDB0002117				**+**																**-**					Conflicting
Fatty Acyls	Oleoylcarnitine, HMDB0005065												**-**				**+**									Conflicting
Fatty Acyls	Stearoylcarnitine, HMDB0062532												**-**				**+**									Conflicting
Glycerophospholipids	LPC (14:0), HMDB0010379			**+**																**-**						Conflicting
Glycerophospholipids	LPC (18:1), HMDB0002815		**+**				**+**					**-**								**-**						Conflicting
Glycerophospholipids	LPC (22:6), HMDB0010404																			**+**	**-**					Conflicting
Glycerophospholipids	PE (18:0/18:1), HMDB0008993																		**-**	**+**						Conflicting
Glycerophospholipids	PC (18:0/18:2), HMDB0008039											**+**								**-**						Conflicting
Glycerophospholipids	PE (18:2/18:0), HMDB0009090																			**-**	**+**					Conflicting
Steroids and Steroid Derivatives	Cholic acid, HMDB0000619																	**-**				**+**				Conflicting
Steroids and Steroid Derivatives	Progesterone, HMDB0001830		**-**		**+**																					Conflicting
Fatty Acyls	Adrenic acid, HMDB0002226									**+**	**-**	**+**								**+**					**+**	Upregulated
Fatty Acyls	Eicosenoic acid, HMDB0002231																			**+**					**+**	Upregulated
Fatty Acyls	Eicosadienoic acid, HMDB0005060																			**+**					**+**	Upregulated
Fatty Acyls	Linoleamide, HMDB0062656		**+**		**+**							**+**														Upregulated
Fatty Acyls	Myristic acid, HMDB0000806				**-**							**+**								**+**						Upregulated
Fatty Acyls	Nervonic acid, HMDB0002368																			**+**					**+**	Upregulated
Fatty Acyls	Palmitaldehyde, HMDB0001551				**+**							**+**														Upregulated
Glycerophospholipids	LPE (16:0), HMDB0011503											**+**								**+**						Upregulated
Glycerophospholipids	PC (16:0/18:1), HMDB0007971																			**+**	**+**					Upregulated
Glycerophospholipids	Platelet-activating factor, HMDB0062195		**+**	**+**																						Upregulated
Sphingolipids	SM (d18:1/18:0), HMDB0001348														**+**					**+**						Upregulated
Steroids and Steroid Derivatives	Cholesterol, HMDB0000067	**-**			**+**																			**+**		Upregulated
Steroids and Steroid Derivatives	Sulfolithocholic acid, HMDB0000907											**+**									**+**					Upregulated

LPC = lysophosphatidylcholine; LPE = lysophosphatidylethanolamine; PC = phosphatidylcholine; PE = phosphatidylethanolamine; SM = sphingomyelin.

**Table 6:** Pre-to-post antipsychotic-treated patients’ lipid signatures. Overall direction is in relation to post-treatment.

Class	Lipid, HMDB ID	Cao 2019 [67]	Kaddurah-Daouk 2007 [59]	Leppik 2020 [27]	Li 2022 [60]	Liu 2021 [66]	Qiao 2016 [29]	Qiu 2023 [65]	Song 2023 [34]	Xuan 2011 [62]	Yan 2018 [31]	Overall Direction
Fatty Acyls	Palmitoleic acid, HMDB0003229		**-**						**-**			Downregulated
Glycerophospholipids	LPC (16:0), HMDB0010382	**-**	**-**						**+**			Downregulated
Glycerophospholipids	LPC (22:6), HMDB0010404					**-**					**-**	Downregulated
Glycerophospholipids	PC (38:6), HMDB0007991							**-**			**-**	Downregulated
Steroids and Steroid Derivatives	CE (22:6), HMDB0245627							**-**			**-**	Downregulated
Fatty Acyls	Docosahexaenoic acid, HMDB0002183		**-**		**+**							Conflicting
Fatty Acyls	Docosapentaenoic acid, HMDB0006528		**-**		**+**							Conflicting
Fatty Acyls	Eicosapentaenoic acid, HMDB0001999		**-**		**+**							Conflicting
Fatty Acyls	Palmitic acid, HMDB0000220		**-**		**+**					**+**	**-**	Conflicting
Fatty Acyls	Stearic acid, HMDB0000827				**+**					**-**		Conflicting
Glycerophospholipids	LPC (15:0), HMDB0010381	**-**				**+**						Conflicting
Glycerophospholipids	LPC (18:2), HMDB0061700		**+**								**-**	Conflicting
Steroids and Steroid Derivatives	CE (20:5), HMDB0006731							**+**			**-**	Conflicting
Fatty Acyls	Adrenic acid, HMDB0002226				**+**				**+**		**-**	Upregulated
Fatty Acyls	Linoleic acid, HMDB0000673				**+**				**-**	**+**		Upregulated
Fatty Acyls	Oleic acid, HMDB0000207				**+**				**-**	**+**		Upregulated
Glycerophospholipids	LPC (14:0), HMDB0010379	**-**	**+**	**+**		**+**	**+**	**+**				Upregulated
Glycerophospholipids	LPC (16:1), HMDB0010383		**+**			**+**						Upregulated
Glycerophospholipids	LPC (18:0), HMDB0010384		**+**			**+**			**+**			Upregulated
Glycerophospholipids	LPC (18:1), HMDB0002815					**+**			**+**			Upregulated
Glycerophospholipids	LPC (20:3), HMDB0010393		**+**	**+**		**+**	**+**	**+**				Upregulated
Steroids and Steroid Derivatives	CE (18:3), HMDB0010369		**+**					**+**				Upregulated

CE = cholesterol ester; FA = fatty acid; LPC = lysophosphatidylcholines; PC = phosphatidylcholines.

## Data Availability

Data sharing is not applicable to this article as no new data were created or analyzed in this study.

## Supplementary Materials

The following supporting information can be downloaded at: https://www.mdpi.com/article/10.3390/metabo14090475/s1, Table S1: Search String. Table S2: Additional characteristics of cross-sectional components of studies examining minimally antipsychotic-treated patients; Table S3: Additional characteristics of cross-sectional components of studies examining antipsychotic-treated patients; Table S4: Additional characteristics of included studies examining pre-to-post antipsychotic-treated patients.
