# Supplementary material for: Peripheral Lipid Signatures, Metabolic Dysfunction, and Pathophysiology in Schizophrenia Spectrum Disorders
Source: Metabolites. 2024 Aug 28;14(9):475. doi: 10.3390/metabo14090475 (PMC11434505; doi:10.3390/metabo14090475)
Supplement: Supplementary file 1 [file metabolites-14-00475-s001.zip › metabolites-3141856-supplementary.pdf]

Supplementary Table S1: Search String

1.	exp "Schizophrenia Spectrum and Other Psychotic Disorders"/
2.	(psychotic disorder* or schizo* or psychos* or psychotic*).tw,kf.
3.	psychosis spectrum.tw,kf.
4.	Antipsychotic free.tw,kf.
5.	Antipsychotic naive.tw,kf.
6.	AP free.tw,kf.
7.	psychotropic free.tw,kf.
8.	drug naive.tw,kf.
9.	drug free.tw,kf.
10.	psychotropic naive.tw,kf.
11.	neuroleptic naive.tw,kf.
12.	neuroleptic free.tw,kf.
13.	untreated.tw,kf.
14.	unmedicated.tw,kf.
15.	un-treated.tw,kf.
16.	un-medicated.tw,kf.
17.	treatment naive.tw,kf.
18.	treatment free.tw,kf.
19.	medication naive.tw,kf.
20.	medication free.tw,kf.
21.	never treated.tw,kf.

22. never medicated.tw,kf.
23. first episode*.tw,kf.
24. exp Metabolome/ and exp Lipidomics/
25. metabolite*.tw,kf.
26. metabotype*.tw,kf.
27. Metabolom*.tw,kf.
28. pharmacometabonomics.tw,kf.
29. lipido*.tw,kf.
30. fluxo*.tw,kf.
31. metabono*.tw,kf.
32. 1 or 2 or 3
33. 4 or 5 or 6 or 7 or 8 or 9 or 10 or 11 or 12 or 13 or 14 or 15 or 16 or 17 or 18 or 19 or 20 or 21 or 22 or 23
34. 24 or 25 or 26 or 27 or 28 or 29 or 30 or 31
35. 32 and 33 and 34
36. limit 35 to (english language and humans)

Supplementary Table S2: Additional characteristics of cross-sectional components of studies examining minimally antipsychotic-treated patients.

Author, year	Country	Targeted vs Untargeted	Biological sample; Fasting	Corrected For Multiple Comparisons	Alcohol, Nicotine and Other Substance Use	Concomitant Medications
Bicikova 2013 # [20]	Czech Republic	NR	Serum; Fasting	Yes	NR	NR
Cai 2012 [23]	China	Targeted	Plasma and Urine; Fasting	Yes	Smoking: Patients = 0.0% HC = 0.0%<br>Excluded: substance use<br>HC screened for alcohol abuse	Alprazolam and benzhexol
Cui 2021 [24]	China	Untargeted	Saliva; Fasting	Yes	Patients = 0.0%, HCs = 3.8%<br>No substance or alcohol use for patients; 1 alcohol use for HCs	NR
Kaddurah-Daouk 2012 [25]	USA	Targeted	Plasma; Fasting	NR	NR	NR
Kriisa 2017 # [26]	Estonia	Targeted	Serum; Fasting	Yes	Smoking: Patients = 21.1%, HCs = 18.9%<br>Cannabis lifetime use = Patients: 26.3%, HC = 2.6%	Antidepressants and mood stabilizers
Lee 2023 [32]	Canada	Targeted	Serum, Fasting	Yes	Smoking: Patients = 20%, HCs = 16.7%<br>HC = 33.3%	NR
Leppik 2020 # [27]	Estonia	Targeted	Serum; Fasting	Yes	Smoking: Patients = 36%, HC = 19%<br>Cannabis use: Patient = 32.1%, HC = 2.7%<br>No participants had cannabis or alcohol dependence	Antidepressants, hypnotics, mood stabilizers
Liu 2014 [38]	China	Targeted	Peripheral blood mononuclear cells; Fasting	NR	Smoking, alcohol: NR<br>Exclusionary: Drug use	NR
Liu 2021 [28]	China	Targeted	Plasma; Fasting	Yes	Smoking: NR<br>Exclusionary: Alcohol abuse and dependence, illicit drug use	NR
Qiao 2016 # [29]	China	NR	Serum; Fasting	NR	Smoking: NR<br>Exclusionary: Alcohol or drug abuse	Benzhexol hydrochloride, benzodiazepines, zolpidem

Schwarz 2011 [30]	Germany	NR	Serum; NR	Yes	Smoking: NR<br>Cannabis consumption present	NR
Shang 2022 *,# [33]	Sweden	Untargeted	Serum; (55% Fasted)	Yes	Smoking: Patients = 22.7%, HCs = 5.0%<br>Alcohol use permitted<br>Substance abuse excluded	Antidepressants, benzodiazepine/ zopiclone, phenothiazine derivatives
Song 2023 # [34]	China	NR	Plasma; Fasting	Yes	NR	NR
Su 2023 [37]	China	Targeted	Plasma, Fasting	Yes	NR	NR
Wang X 2024 * [36]	China	Targeted	Plasma, Fasting	No	Smoking: Patients = 7.90%, HCs = 0.0%<br>Exclusionary: Alcoholism or other substance abuse disorders	Benzodiazepines
Wang Z 2024 [35]	China	Untargeted	Serum, Fasting	Yes	Smoking: Patients = 2.4%, HCs = 2.4%<br>Substance abuse and alcohol NR	NR
Yan 2018 # [31]	China	Untargeted	Plasma; Fasting	Yes	Patients and HCs abstained from alcohol or smoking	NR

HCs = healthy controls; NR = not reported; * = shared studies between minimally AP-treated and AP-treated groups; # = shared studies between minimally AP-treated and pre-to-post AP treatment groups.

Supplementary Table S3: Additional characteristics of cross-sectional components of studies examining antipsychotic-treated patients.

Author, year;	Country	Targeted vs Untargeted	Biological sample; Fasting	Corrected for Multiple Comparisons	Alcohol, Nicotine and Other Substance Use	Concomitant Medications
AlAwam 2015 [39]	Germany	NR	Serum; NR	Yes	Smokers: Patients = 53.8%, HCs = NR<br>Alcohol and other substances NR	NR
Avigdor 2021 [40]	USA	Targeted	Plasma; NR	Yes	Smoking: Patients = 29%, HCs = 5.7%<br>Exclusion criteria: no drug or alcohol abuse in the past three years; and no illicit drug use in the past two months	Antidepressants
Campeau 2022 [41]	USA	Untargeted	Plasma; Fasting	Yes	Smoking: Patients = 55.6%, HCs = 0.0%<br>Substance use: Patients: 48.1%, HCs = 11.1%<br>Alcohol abuse: Patients: 44.4%, HCs = 22.0%	NR
Cui 2020 [42]	China	Untargeted	Serum; Fasting	Yes	Smoking: Patients = 16.7%, HCs = 14.8 %<br>Exclusionary: alcohol abuse<br>NR for other substances	Mood stabilizing drugs in the past 2 weeks excluded
Dickens 2020 [43]	Finland and London	Targeted	Serum; NR	NR	Smoking: NR<br>Exclusionary: Current/lifetime history of substance abuse/dependence or substance abuse within the last month<br>Cannabis dependence disorder: 12.5%	NR
Du 2021 [44]	China	Targeted	Blood exosome; Fasting	NR	Smoking: NR<br>Exclusionary: substance abuse	NR
Fukushima 2014 [45]	Japan	Targeted	Serum; NR	Yes	Smoking: Patients = 40%, HCs = 3.7%<br>Alcohol and other substances NR	NR

He 2012 [46]	Germany	Targeted	Plasma; NR	Yes	NR	NR
Kaddurah-Daouk 2007 # [59]	USA	NR	Plasma; Fasting	NR	Patients continued smoking<br>Exclusionary: psychoactive substance abuse	NR
Koike 2014 [47]	Japan	NR	Plasma; NR	NR	Smoking: Patient = 11.1 %, HCs = 7.1%<br>Exclusionary: history of alcohol addiction, illegal substance use	Benzodiazepines
Li 2022 # [60]	China	Targeted	Erythrocyte membranes; Yes	Yes	Smoking: NR<br>Exclusionary: Alcohol or drug abuse	NR
Liu 2020 [48]	China	NR	Serum; Fasting	Yes	Smoking: NR<br>Exclusionary: substance abuse	NR
Mednova 2021 [49]	Russia	NR	Serum; Fasting	NR	NR	No valproic acid and no antidiabetic drugs
Oresic 2011 [50]	Finland	NR	Serum; Fasting	NR	Smoking: Patients = 44.4%, HCs = 26.7 %<br>Alcohol and substance abuse NR	Antidiabetic medications
Oresic 2012 [51]	Finland	NR	Plasma; Fasting	Yes	NR	Cholesterol medication
Paredes 2014 [52]	USA	NR	Plasma; Fasting	Yes	NR	Analgesic, anticonvulsant, antidyskinetic, antihistaminic, blood pressure, mood regulation, sleep aid
Parksepp 2022 [64]	Estonia	Targeted	Serum, Fasting	Yes	Smoking: Patients = 37%, HCs = 26%<br>Cannabis use: Patients 35%, HCs = 26%	Antidepressants, benzodiazepines, hypnotics or mood stabilizers
Qing 2022 [63]	China	Targeted	Serum; Fasting	Yes	Smoking: Patients = 25.4%, HCs = 20.0%<br>Exclusionary: heavy consumption of alcohol	Mood stabilizing drugs in the past 2 weeks excluded
Shang 2022 *,# [33]	Sweden	Untargeted	Serum, 55% Fasting	Yes	Smoking: Patients = 22.7%, HCs = 5.0%<br>Exclusionary: substance abuse	Antidepressants

					Alcohol use was permitted	
Tasic 2017 [53]	Brazil	NR	Serum; Fasting	NR	Smoking: NR<br>Exclusionary: current substance use disorder	NR
Tasic 2019 [54]	Brazil	NR	Serum; Fasting	NR	Smoking: Patients = 28%, HCs = 11.6%<br>Exclusionary: substance abuse or drug dependence	Clonazepam, Fluoxetine, Lithium, Valproic acid
Tessier 2016 [55]	Belgium	NR	RBC membrane; Fasting	NR	NR	NR
Wang 2021 [61]	China	Targeted	Serum, Fasting	Yes	Smoking: Patients = 11.8 %, HC = 4.6%<br>Alcohol use: Patients = 6.7%, HCs = 14.7%<br>Exclusionary: alcohol and substance abuse disorders	NR
Wang 2022 [56]	China	Untargeted	Plasma; Fasting	Yes	Smoking: NR<br>Exclusionary: substance abuse	NR
Wang X 2024 * [36]	China	Targeted	Plasma; Fasting	NR	Smoking: Patient = 7.89%, HC = 0.0%<br>Exclusionary: alcohol or substance abuse disorders	Benzodiazepines
Wood 2015 [57]	USA	NR	Plasma and platelets; Fasting	NR	NR	NR
Xuan 2011 # [62]	China	NR	Serum, Fasting	NR	Abstained from smoking and alcohol<br>Exclusionary: alcohol and substance abuse	NR
Yang 2017 [58]	China	Targeted	Serum; Fasting	Yes	Smoking and substance abuse: NR<br>Exclusionary: alcohol abuse	NR

HCs = healthy controls; NR = not reported; RBC = red blood cell; * = shared studies between minimally AP-treated and AP-treated groups; # = shared studies between AP-treated and pre-to-post AP treatment groups.

Supplementary Table S4: Additional characteristics of included studies examining pre-to-post AP-treated patients.

Author, Year	Country	Targeted Vs Untargeted	Biological sample; Fasting	Corrected for Multiple Comparisons	Alcohol, Nicotine and Other Substance Use	Concomitant Medications
Bicikova 2013* [20]	Czech Republic	NR	Serum; Fasting	Yes	NR	NR
Cao 2019 [67]	China	Untargeted	Serum; Fasting	Yes	Abstained from smoking and alcohol<br>Exclusionary: alcohol and drug use disorders	Anxiolytics, anticonvulsants, anti-tremor medications
Kaddurah-Daouk 2007# [59]	USA	NR	Plasma; Fasting	Yes	Patients continued smoking<br>Exclusionary: psychoactive substance abuse	NR
Kriisa 2017* [26]	Estonia	Targeted	Serum; Fasting	Yes	Smoking: 21.1%<br>Cannabis lifetime use: 26.3%	Antidepressants and mood stabilizers
Leppik 2020* [27]	Estonia	Targeted	Serum; Fasting	Yes	Smoking: 36%<br>Cannabis use: 32.1%<br>No participants had cannabis or alcohol dependence	Antidepressants, mood stabilizers, or hypnotics
Li 2022# [60]	China	Targeted	Erythrocyte Membrane; Fasting	NR	Smoking: NR<br>Exclusionary: alcohol or drug abuse	NR

Liu 2021 [66]	China	Untargeted	Plasma, Fasting	Yes	Exclusionary: substance abuse, including alcohol and smoking	NR
Qiao 2016* [29]	China	NR	Serum; Fasting	NR	Smoking: NR Exclusionary: alcohol or drug abuse	Benzhexol, benzodiazepines, hydrochloride zolpidem
Qiu 2023 [65]	China	Targeted	Plasma; Fasting	Yes	Smoking: 23.3% Exclusionary: Alcohol or substance abuse	Benzodiazepines
Shang 2022* [33]	Sweden	Untargeted	Serum; Fasting	Yes	Smoking: 22.7% Alcohol use permitted Exclusionary: substance abuse	Antidepressants, benzodiazepine/ zopiclone, phenothiazine derivatives
Song 2023* [34]	China	NR	Plasma, Fasting	Yes	NR	NR
Xuan 2011# [62]	China	NR	Serum; Fasting	NR	Abstained from smoking and alcohol Exclusionary: alcohol and substance abuse	NR
Yan 2018* [31]	China	Untargeted	Plasma; Fasting	Yes	Abstained from smoking and alcohol	NR

HCs = healthy controls; NR = not reported; * = shared studies between minimally AP-treated and AP-treated groups; # = shared studies between AP-treated and pre-to-post AP treatment groups.

References

1. Bicikova, M.; Hill, M.; Ripova, D.; Mohr, P.; Hampl, R. Determination of Steroid Metabolome as a Possible Tool for Laboratory Diagnosis of Schizophrenia. *J. Steroid Biochem. Mol. Biol.* **2013**, *133*, 77–83. https://doi.org/10.1016/j.jsbmb.2012.08.009.
2. Wishart, D.S.; Guo, A.C.; Oler, E.; Wang, F.; Anjum, A.; Peters, H.; Dizon, R.; Sayeeda, Z.; Tian, S.; Lee, B.L.; et al. HMDB 5.0: The Human Metabolome Database for 2022. *Nucleic Acids Res* **2022**, *50*, D622–D631. https://doi.org/10.1093/nar/gkab1062.
3. Moola, S.; Munn, Z.; Tufanaru, C.; Aromataris, E.; Sears, K.; Sfetcu, R.; Currie, M.; Qureshi, R.; Mattis, P.; Lisy, K.; et al. Chapter 7: Systematic Reviews of Etiology and Risk Available online: https://synthesismanual.jbi.global (accessed on 14 April 2023).
4. Cai, H.L.; Li, H. De; Yan, X.Z.; Sun, B.; Zhang, Q.; Yan, M.; Zhang, W.Y.; Jiang, P.; Zhu, R.H.; Liu, Y.P.; et al. Metabolomic Analysis of Biochemical Changes in the Plasma and Urine of First-Episode Neuroleptic-Naïve Schizophrenia Patients after Treatment with Risperidone. *J. Proteome Res.* **2012**, *11*, 4338–4350. https://doi.org/10.1021/pr300459d.
5. Cui, G.; Qing, Y.; Li, M.; Sun, L.; Zhang, J.; Feng, L.; Li, J.; Chen, T.; Wang, J.; Wan, C. Salivary Metabolomics Reveals That Metabolic Alterations Precede the Onset of Schizophrenia. *J. Proteome Res.* **2021**, *20*, 5010–5023. https://doi.org/10.1021/acs.jproteome.1c00504.
6. Kaddurah-Daouk, R.; Mcevoy, J.; Baillie, R.; Zhu, H.; Yao, J.K.; Nimgaonkar, V.L.; Buckley, P.F.; Keshavan, M.S.; Georgiades, A.; Nasrallah, H.A. Impaired Plasmalogens in Patients with Schizophrenia. *Psychiatry Res.* **2012**, *198*, 347–352. https://doi.org/10.1016/j.psychres.2012.02.019.
7. Kriisa, K.; Leppik, L.; Balõtšev, R.; Ottas, A.; Soomets, U.; Koido, K.; Volke, V.; Rgen Innos, J.; Haring, L.; Vasar, E.; et al. Profiling of Acylcarnitines in First Episode Psychosis before and after Antipsychotic Treatment. *J. Proteome Res.* **2017**, *16*, 3558–3566. https://doi.org/10.1021/acs.jproteome.7b00279.
8. Leppik, L.; Parksepp, M.; Janno, S.; Koido, K.; Haring, L.; Vasar, E.; Zilmer, M. Profiling of Lipidomics before and after Antipsychotic Treatment in First-Episode Psychosis. *Eur. Arch. Psychiatry Clin. Neurosci.* **2020**, *270*, 59–70. https://doi.org/10.1007/s00406-018-0971-6.
9. Liu, Y.; Song, X.; Liu, X.; Pu, J.; Gui, S.; Xu, S.; Tian, L.; Zhong, X.; Zhao, L.; Wang, H.; et al. Alteration of Lipids and Amino Acids in Plasma Distinguish Schizophrenia Patients from Controls: A Targeted Metabolomics Study. *Psychiatry Clin. Neurosci.* **2021**, *75*, 138–144. https://doi.org/10.1111/PCN.13194.
10. Qiao, Y.; Zhang, L.; He, S.; Wen, H.; Yu, Y.-M.; Cao, C.-H.; Li, H.-F. Plasma Metabonomics Study of First-Episode Schizophrenia Treated with Olanzapine in Female Patients. *Neurosci. Lett.* **2016**, *617*, 270–276. https://doi.org/10.1016/j.neulet.2016.02.031.
11. Schwarz, E.; Whitfield, P.; Nahnsen, S.; Wang, L.; Major, H.; Leweke, F.M.; Koethe, D.; Lio, P.; Bahn, S. Alterations of Primary Fatty Acid Amides in Serum of Patients with Severe Mental Illness. *Front. Biosci. (Elite Ed)* **2011**, *3*, 308–314. https://doi.org/10.2741/e246.
12. Yan, L.; Zhou, J.; Wang, D.; Si, D.; Liu, Y.; Zhong, L.; Yin, Y.; Yan, L. Unbiased Lipidomic Profiling Reveals Metabolomic Changes during the Onset and Antipsychotics Treatment of Schizophrenia Disease. *Metabolomics* **2018**, *14*, 80. https://doi.org/10.1007/s11306-018-1375-3.
13. Lee, J.; Costa-Dookhan, K.; Panganiban, K.; MacKenzie, N.; Treen, Q.C.; Chintoh, A.; Remington, G.; Müller, D.J.; Sockalingam, S.; Gerretsen, P.; et al. Metabolomic Signatures Associated with Weight Gain and Psychosis Spectrum Diagnoses: A Pilot Study. *Front. Psychiatry* **2023**, *14*, 1169787. https://doi.org/10.3389/fpsyt.2023.1169787.
14. Shang, P.; Man-Choi Ho, A.; Tufvesson-Alm, M.; Lindberg, D.R.; Grant, C.W.; Orhan, F.; Eren, F.; Bhat, M.; Engberg, G.; Schwieler, L.; et al. Identification of Cerebrospinal Fluid and Serum Metabolomic Biomarkers in First Episode Psychosis Patients. *Transl. Psychiatry* **2022**, *12*, 229. https://doi.org/10.1038/s41398-022-02000-1.
15. Song, M.; Liu, Y.; Zhou, J.; Shi, H.; Su, X.; Shao, M.; Yang, Y.; Wang, X.; Zhao, J.; Guo, D.; et al. Potential Plasma Biomarker Panels Identification for the Diagnosis of First-Episode Schizophrenia and Monitoring Antipsychotic Monotherapy with the Use of Metabolomics Analyses. *Psychiatry Res.* **2023**, *321*, 115070. https://doi.org/10.1016/j.psychres.2023.115070.
16. Wang, Z.; Yuan, X.; Zhu, Z.; Pang, L.; Ding, S.; Li, X.; Kang, Y.; Hei, G.; Zhang, L.; Zhang, X.; et al. Multiomics Analyses Reveal Microbiome-Gut-Brain Crosstalk Centered on Aberrant Gamma-Aminobutyric Acid and Tryptophan Metabolism in Drug-Naïve Patients with First-Episode Schizophrenia. *Schizophr. Bull.* **2024**, *50*, 187–198. https://doi.org/10.1093/schbul/sbad026.
17. Wang, X.; Xie, J.; Ma, H.; Li, G.; Li, M.; Li, S.; Sun, X.; Zhao, Y.; Sun, W.; Yang, S.; et al. The Relationship between Alterations in Plasma Metabolites and Treatment Responses in Antipsychotic-Naïve Female Patients with Schizophrenia. *World J. Biol. Psychiatry* **2024**, *25*, 106–115.

18. Su, Q.; Bi, F.; Yang, S.; Yan, H.; Sun, X.; Wang, J.; Qiu, Y.; Li, M.; Li, S.; Li, J. Identification of Plasma Biomarkers in Drug-Naïve Schizophrenia Using Targeted Metabolomics. *Psychiatry Investig.* **2023**, *20*, 818–825. https://doi.org/10.30773/pi.2023.0121.
19. Liu, M.L.; Zheng, P.; Liu, Z.; Xu, Y.; Mu, J.; Guo, J.; Huang, T.; Meng, H.Q.; Xie, P. GC-MS Based Metabolomics Identification of Possible Novel Biomarkers for Schizophrenia in Peripheral Blood Mononuclear Cells. *Mol. Biosyst.* **2014**, *10*, 2398–2406. https://doi.org/10.1039/c4mb00157e.
20. Al Awam, K.; Haußleiter, I.S.; Dudley, E.; Donev, R.; Brüne, M.; Juckel, G.; Thome, J. Multiplatform Metabolome and Proteome Profiling Identifies Serum Metabolite and Protein Signatures as Prospective Biomarkers for Schizophrenia. *J. Neural Transm.* **2015**, *122*, 111–122. https://doi.org/10.1007/s00702-014-1224-0.
21. Avigdor, B.E.; Yang, K.; Shinder, I.; Orsburn, B.C.; Rais, R.; Kano, S.I.; Sawa, A.; Pevsner, J. Characterization of Antipsychotic Medications, Amino Acid Signatures, and Platelet-Activating Factor in First-Episode Psychosis. *Biomark. Neuropsychiatry* **2021**, *5*, 100045. https://doi.org/10.1016/j.bionps.2021.100045.
22. Campeau, A.; Mills, R.H.; Stevens, T.; Rossitto, L.A.; Meehan, M.; Dorrestein, P.; Daly, R.; Nguyen, T.T.; Gonzalez, D.J.; Jeste, D.V.; et al. Multi-Omics of Human Plasma Reveals Molecular Features of Dysregulated Inflammation and Accelerated Aging in Schizophrenia. *Mol. Psychiatry* **2022**, *27*, 1217–1225. https://doi.org/10.1038/s41380-021-01339-z.
23. Cui, G.; Qing, Y.; Hu, X.; Wang, P.; Sun, L.; Yang, X.; Jiang, J.; Zhang, J.; Wang, H.; Feng, L.; et al. Serum Metabolomic Profiling Based on Fourier Transform-Ion Cyclotron Resonance-Mass Spectrometry: Do the Dysfunctions of Metabolic Pathways Reveal a Universal Risk of Oxidative Stress in Schizophrenia? *Antioxid. Redox Signal.* **2020**, *33*, 679–688.
24. Dickens, A.M.; Borgan, F.; Laurikainen, H.; Lamichhane, S.; Marques, T.; Rönkkö, T.; Veronese, M.; Lindeman, T.; Hyötyläinen, T.; Howes, O.; et al. Links between Central CB1-Receptor Availability and Peripheral Endocannabinoids in Patients with First Episode Psychosis. *NPJ Schizophr.* **2020**, *6*, 21. https://doi.org/10.1038/s41537-020-00110-7.
25. Du, Y.; Chen, L.; Li, X.S.; Li, X.L.; Xu, X.D.; Tai, S.B.; Yang, G.L.; Tang, Q.; Liu, H.; Liu, S.H.; et al. Metabolomic Identification of Exosome-Derived Biomarkers for Schizophrenia: A Large Multicenter Study. *Schizophr. Bull.* **2021**, *47*, 615–623. https://doi.org/10.1093/schbul/sbaa166.
26. Fukushima, T.; Iizuka, H.; Yokota, A.; Suzuki, T.; Ohno, C.; Kono, Y.; Nishikiori, M.; Seki, A.; Ichiba, H.; Watanabe, Y.; et al. Quantitative Analyses of Schizophrenia-Associated Metabolites in Serum: Serum D-Lactate Levels Are Negatively Correlated with Gamma-Glutamylcysteine in Medicated Schizophrenia Patients. *PLoS ONE* **2014**, *9*, e101652. https://doi.org/10.1371/journal.pone.0101652.
27. He, Y.; Yu, Z.; Giegling, I.; Xie, L.; Hartmann, A.M.; Prehn, C.; Adamski, J.; Kahn, R.; Li, Y.; Illig, T.; et al. Schizophrenia Shows a Unique Metabolomics Signature in Plasma. *Transl. Psychiatry* **2012**, *2*, e149. https://doi.org/10.1038/TP.2012.76.
28. Koike, S.; Bundo, M.; Iwamoto, K.; Suga, M.; Kuwabara, H.; Ohashi, Y.; Shinoda, K.; Takano, Y.; Iwashiro, N.; Satomura, Y.; et al. A Snapshot of Plasma Metabolites in First-Episode Schizophrenia: A Capillary Electrophoresis Time-of-Flight Mass Spectrometry Study. *Transl. Psychiatry* **2014**, *4*, e379. https://doi.org/10.1038/tp.2014.19.
29. Liu, L.; Zhao, J.; Chen, Y.; Feng, R. Metabolomics Strategy Assisted by Transcriptomics Analysis to Identify Biomarkers Associated with Schizophrenia. *Anal. Chim. Acta* **2020**, *1140*, 18–29. https://doi.org/10.1016/J.ACA.2020.09.054.
30. Mednova, I.A.; Chernonosov, A.A.; Kasakin, M.F.; Kornetova, E.G.; Semke, A.V.; Bokhan, N.A.; Koval, V.V.; Ivanova, S.A. Amino Acid and Acylcarnitine Levels in Chronic Patients with Schizophrenia: A Preliminary Study. *Metabolites* **2021**, *11*, 34. https://doi.org/10.3390/metabo11010034.
31. Orešič, M.; Tang, J.; Seppänen-Laakso, T.; Mattila, I.; Saarni, S.E.; Saarni, S.I.; Lönnqvist, J.; Sysi-Aho, M.; Hyötyläinen, T.; Perälä, J.; et al. Metabolome in Schizophrenia and Other Psychotic Disorders: A General Population-Based Study. *Genome Med.* **2011**, *3*, 1–14. https://doi.org/10.1186/GM233/FIGURES/3.
32. Orešič, M.; Seppänen-Laakso, T.; Sun, D.; Tang, J.; Therman, S.; Viehman, R.; Mustonen, U.; van Erp, T.G.; Hyötyläinen, T.; Thompson, P.; et al. Phospholipids and Insulin Resistance in Psychosis: A Lipidomics Study of Twin Pairs Discordant for Schizophrenia. *Genome Med.* **2012**, *4*, 1. https://doi.org/10.1186/gm300.
33. Paredes, R.M.; Quinones, M.; Marballi, K.; Gao, X.; Valdez, C.; Ahuja, S.S.; Velligan, D.; Walss-Bass, C. Metabolomic Profiling of Schizophrenia Patients at Risk for Metabolic Syndrome. *Int. J. Neuropsychopharmacol.* **2014**, *17*, 1139–1148. https://doi.org/10.1017/S1461145714000157.

34. Tasic, L.; Pontes, J.G.M.; Carvalho, M.S.; Cruz, G.; Dal Mas, C.; Sethi, S.; Pedrini, M.; Rizzo, L.B.; Zeni-Graiff, M.; Asevedo, E.; et al. Metabolomics and Lipidomics Analyses by 1H Nuclear Magnetic Resonance of Schizophrenia Patient Serum Reveal Potential Peripheral Biomarkers for Diagnosis. *Schizophr. Res.* **2017**, *185*, 182–189. https://doi.org/10.1016/j.schres.2016.12.024.
35. Tasic, L.; Larcerda, A.L.T.; Pontes, J.G.M.; da Costa, T.B.B.C.; Nani, J.V.; Martins, L.G.; Santos, L.A.; Nunes, M.F.Q.; Adelino, M.P.M.; Pedrini, M.; et al. Peripheral Biomarkers Allow Differential Diagnosis between Schizophrenia and Bipolar Disorder. *J. Psychiatr. Res.* **2019**, *119*, 67–75. https://doi.org/10.1016/j.jpsychires.2019.09.009.
36. Tessier, C.; Sweers, K.; Frajerman, A.; Bergaoui, H.; Ferreri, F.; Delva, C.; Lapidus, N.; Lamaziere, A.; Roiser, J.P.; De Hert, M.; et al. Membrane Lipidomics in Schizophrenia Patients: A Correlational Study with Clinical and Cognitive Manifestations. *Transl. Psychiatry* **2016**, *6*, e906. https://doi.org/10.1038/tp.2016.142.
37. Wang, T.; Li, P.; Meng, X.; Zhang, J.; Liu, Q.; Jia, C.; Meng, N.; Zhu, K.; Lv, D.; Sun, L.; et al. An Integrated Pathological Research for Precise Diagnosis of Schizophrenia Combining LC-MS/1H NMR Metabolomics and Transcriptomics. *Clinica Chimica Acta* **2022**, *524*, 84–95. https://doi.org/10.1016/j.cca.2021.11.028.
38. Wood, P.L.; Unfried, G.; Whitehead, W.; Phillipps, A.; Wood, J.A. Dysfunctional Plasmalogen Dynamics in the Plasma and Platelets of Patients with Schizophrenia. *Schizophr. Res.* **2015**, *161*, 506–510. https://doi.org/10.1016/j.schres.2014.11.032.
39. Yang, X.; Sun, L.; Zhao, A.; Hu, X.; Qing, Y.; Jiang, J.; Yang, C.; Xu, T.; Wang, P.; Liu, J.; et al. Serum Fatty Acid Patterns in Patients with Schizophrenia: A Targeted Metabonomics Study. *Transl. Psychiatry* **2017**, *7*, e1176. https://doi.org/10.1038/tp.2017.152.
40. Kaddurah-Daouk, R.; McEvoy, J.; Baillie, R.A.; Lee, D.; Yao, J.K.; Doraiswamy, P.M.; Krishnan, K.R.R. Metabolomic Mapping of Atypical Antipsychotic Effects in Schizophrenia. *Mol. Psychiatry* **2007**, *12*, 934–945. https://doi.org/10.1038/sj.mp.4002000.
41. Li, N.; Yang, P.; Tang, M.; Liu, Y.; Guo, W.; Lang, B.; Wang, J.; Wu, H.; Tang, H.; Yu, Y.; et al. Reduced Erythrocyte Membrane Polyunsaturated Fatty Acid Levels Indicate Diminished Treatment Response in Patients with Multi- versus First-Episode Schizophrenia. *Schizophrenia* **2022**, *8*, 7. https://doi.org/10.1038/s41537-022-00214-2.
42. Wang, D.; Sun, X.; Maziade, M.; Mao, W.; Zhang, C.; Wang, J.; Cao, B. Characterising Phospholipids and Free Fatty Acids in Patients with Schizophrenia: A Case-Control Study. *World Journal of Biological Psychiatry* **2021**, *22*, 161–174. https://doi.org/10.1080/15622975.2020.1769188.
43. Xuan, J.; Pan, G.; Qiu, Y.; Yang, L.; Su, M.; Liu, Y.; Chen, J.; Feng, G.; Fang, Y.; Jia, W.; et al. Metabolomic Profiling to Identify Potential Serum Biomarkers for Schizophrenia and Risperidone Action. *J. Proteome Res.* **2011**, *10*, 5433–5443. https://doi.org/10.1021/pr2006796.
44. Qing, Y.; Wang, P.; Cui, G.; Zhang, J.; Liang, K.; Xia, Z.; Wang, P.; He, L.; Jia, W. Targeted Metabolomics Reveals Aberrant Profiles of Serum Bile Acids in Patients with Schizophrenia. *Schizophrenia* **2022**, *8*, 65. https://doi.org/10.1038/s41537-022-00273-5.
45. Parksepp, M.; Haring, L.; Kilk, K.; Taalberg, E.; Kangro, R.; Zilmer, M.; Vasar, E. A Marked Low-Grade Inflammation and a Significant Deterioration in Metabolic Status in First-Episode Schizophrenia: A Five-Year Follow-Up Study. *Metabolites* **2022**, *12*, 983. https://doi.org/10.3390/metabo12100983.
